# Bibliometric study of the systematic reviews and meta-analyses in dentistry

**DOI:** 10.4317/jced.60884

**Published:** 2023-11-01

**Authors:** Beatriz Tarazona-Álvarez, Andrés López-Roldán, Antonio Vidal-Infer, Adolfo Alonso-Arroyo

**Affiliations:** 1 Department of Stomatology. School of Medicine and Dentistry, University of Valencia, Spain; 2 Department of History of Science and Information Science, School of Medicine and Dentistry, University of Valencia, Spain; 3 UISYS, Joint Research Unit (CSIC – University of Valencia)

## Abstract

**Background:**

Scientific production has increased in the last decades, consequently the number of systematic reviews, reviews and meta-analyses, the objective is to carry out a bibliometric analysis study of systematic reviews and meta-analyses in dentistry, divided into different thematic areas.

**Material and Methods:**

A search was conducted in the Science Citation Index-Expanded on the core collection of Web of Science, they were selected from the area of Dentistry and Oral Surgery and Medicine categories, the data was downloaded on April 20, 2022 and The 100 most cited articles from each of the thematic areas were selected.

**Results:**

An increase in this type of articles was observed in the last decade in the areas of pediatric and medical dentistry and oral pathology. The thematic area that received the most citations was periodontics. The two authors with the most citations are Zwahlen, Marcel and Pjetursson, Bjarni Eluar. The countries that receive the most citations are European, along with the USA and China. The topics that are most published in this type of article deal with Cancer with 50 publications, caries treatment with 25 and fluoridation with 1. The entities that finance this type of articles the most are private companies (26.76%).

**Conclusions:**

Together with an exponential increase in the number of publications in dentistry, there has been an increase in the number of publications in systematic reviews. The areas publishing the most articles and having the most citations are Periodontics and Implantology, despite the fact that the most studied topic is cancer.

** Key words:**Bibliometrics, methodological study, systematics reviews, metanalys.

## Introduction

Scientific research has increased considerably in the recent decades, leading to an increased number of publications. This growing evolution of research warrants critical evaluation that helps enhance its results in both quantity and quality.

Bibliometric analysis is defined as the application of mathematics and statistical methods to scientific production. The most important bibliometric indicators are the impact factor, number of citations, or relevant data such as affiliation, authorship, geographical distribution, and funding sources (1).

In the field of dentistry, many bibliometric studies have been conducted either in the general scope (2) or in thematic areas, such as Periodontics (3), Orthodontics (4), Implantology (5), Endodontics (6), and Prosthodontics(7). Furthermore, certain aspects, such as pathologies, treatments, and specific materials, have been analysed (8-11).

In this analysis of scientific production, a progressive increase in systematic reviews, with or without meta-analyses, as well as literature reviews has been observed (2). This growth can be attributed to the importance of these works as tools to analyse existing evidence (12). Systematic reviews allow summarising existing knowledge and represent a way of applying evidence-based dentistry to clinical practice(13).

Owing to the large number of such publications and their scientific relevance, it is interesting to analyse this type of scientific production from a bibliometric point of view. To date, only few studies have addressed specific aspects of the different clinical areas of dentistry (14).

## Material and Methods

-Search strategy

A search was conducted in the Science Citation Index-Expanded on the core collection of Web of Science (referencia Clarivate Analytics), since it is commonly used in bibliometric studies because of its wide thematic coverage and the possibility of counting citations of the articles and assessing the participating institutions in each work.

The search strategy was performed in the topic field (title, keywords, and abstract) with the following terms: (TS=(“metaanaly*” OR “meta-analy*” OR “metanaly*” OR “meta-overview*” OR “metareview*“ OR “meta-review*“ OR “metasynthes*“ OR “meta-synthes*“ OR “systematic* analytical review*” OR “systematic* and critical review*” OR “systematic* and other review*” OR “systematic* descriptive review*” OR “systematic* epidemiological review*” OR “systematic* evidence review*” OR “systematic* imaging review*” OR “systematic* integration literature review*” OR “systematic* literature and case review*” OR “systematic* literature review*“ OR “systematic* mapping review*” OR “systematic* meta-analysis review*” OR “systematic* multivocal literature review*” OR “systematic* narrative review*” OR “systematic* overview*“ OR “systematic* qualitative review*” OR “systematic* quantitative review*” OR “systematic* research projects review*” OR “systematic* research review*” OR “systematic* review*“ OR “systematic* scoping review*” OR “systematic* treatment review*” OR “systematic* evidence-based review*” OR “systematic* retrospective review*”)) AND (DT==(“ARTICLE” OR “REVIEW”) AND TASCA==(“DENTISTRY ORAL SURGERY MEDICINE”)).

Article and review document typologies were selected in the Dentistry and Oral Surgery and Medicine categories, and the download was performed on 20 April 2022. After executing the search, 7,595 records were retrieved. From the download, the titles were analysed to classify them into the following categories: Paediatric dentistry, Orthodontics, Periodontics, Implantology, Endodontics, Oral and Maxillofacial surgery, and Prosthodontics. This was done until we got the 100 most-cited articles in each of the categories. In some specialties, 101 articles were obtained, because they had the same number of citations.

-Data normalisation

Manual normalisation of the records was performed. The normalisation process comprised identifying all the variants of the same name or institution and assigning each a unique identification. Authors, institutions, countries, journals, keywords, and funding institutions were normalised to obtain results.

In the normalisation of the authors, 2,812 author signatures were identified. The main problems in normalisation were related to the existing differences in the degree of development of names and surnames, i.e. for the same author, different variants were found depending on the information provided. In such cases, the institutional affiliations that appeared in the database from which the records were extracted were consulted. If the information could not be obtained in this way, a search was conducted on the websites of the institutions to solve possible conflicts. When there were several variants for the same author, it was considered as the same person if their institutional affiliation also coincided, and the signature that provided the most information in all the cases was used.

Regarding institutions, 708 institutional signatures were initially found, and the criteria followed were similar to those used for authorship. However, in this instance and in accordance with the objectives of this study, only the macro-institutions were used, i.e. universities, research centres or institutes, foundations, hospitals, and others. In case of doubtful data with incomplete information or imprecise or erroneous use of abbreviations or organisational subdivisions, such as faculties, departments, and units, the original databases were consulted. If the information could not be retrieved that way, a search on the Internet was performed to obtain the relevant data. Once the normalisation process was performed, duplicates were eliminated. When there was no affiliation to a specific institution, the label ‘Independent’ was assigned, considering that it was the workplace of self-employed people. Additionally, analysis of the most productive funding sources and most frequent keywords was conducted for the articles.

-Data analysis

To study scientific production, the temporal evolution of scientific productivity, authors, institutions, and countries and journals where the articles were published, as well as keywords and funding sources were examined.

The records were imported into a Microsoft Access (Microsoft, Alburquerque USA) database for debugging and analysis of the results. Graphical representations of the clusters of authors, institutions, and keywords were constructed using the open-source network analysis and visualisation software Pajek.

## Results

Table 1 shows the evolution of the published articles by decades and specialties. An increase in publications over time can be observed in specialties such as Paediatric dentistry or Medicine and Oral Pathology; however, the number of publications in Periodontics and Implantology in the last two decades has been constant.

**Table 1:**
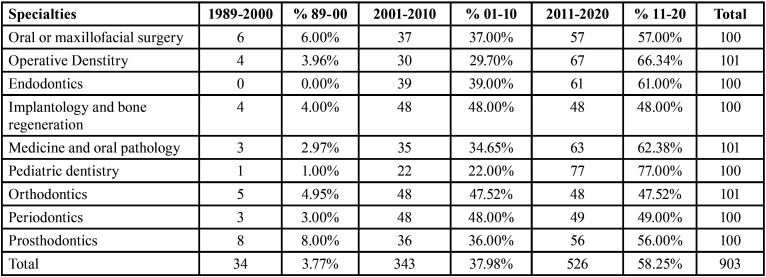
Evolution of published articles distributed by decades and specialties.

Table 2 shows the average number of citations per published article, highlighting that the specialties of Periodontics and Implantology have the most number of citations, which is considerably higher than that of the other specialties (more than 200 citations).

**Table 2:**
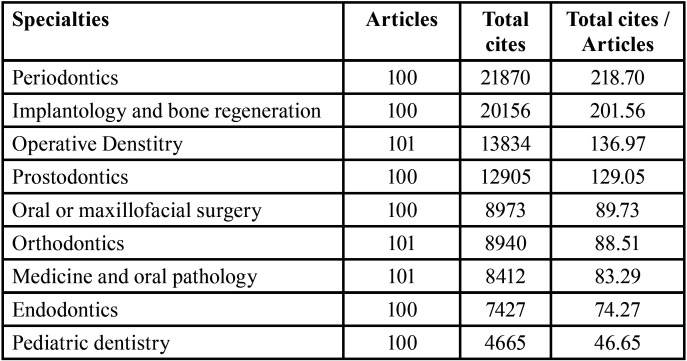
Number of citations and average citations per article in each of the specialties.

Table 3 presents the most productive authors of systematic reviews and/or meta-analyses. All of them have published studies in different specialties, which is in contrast to other disciplines where the authors usually specialise only in one specialty of dentistry.

**Table 3:**
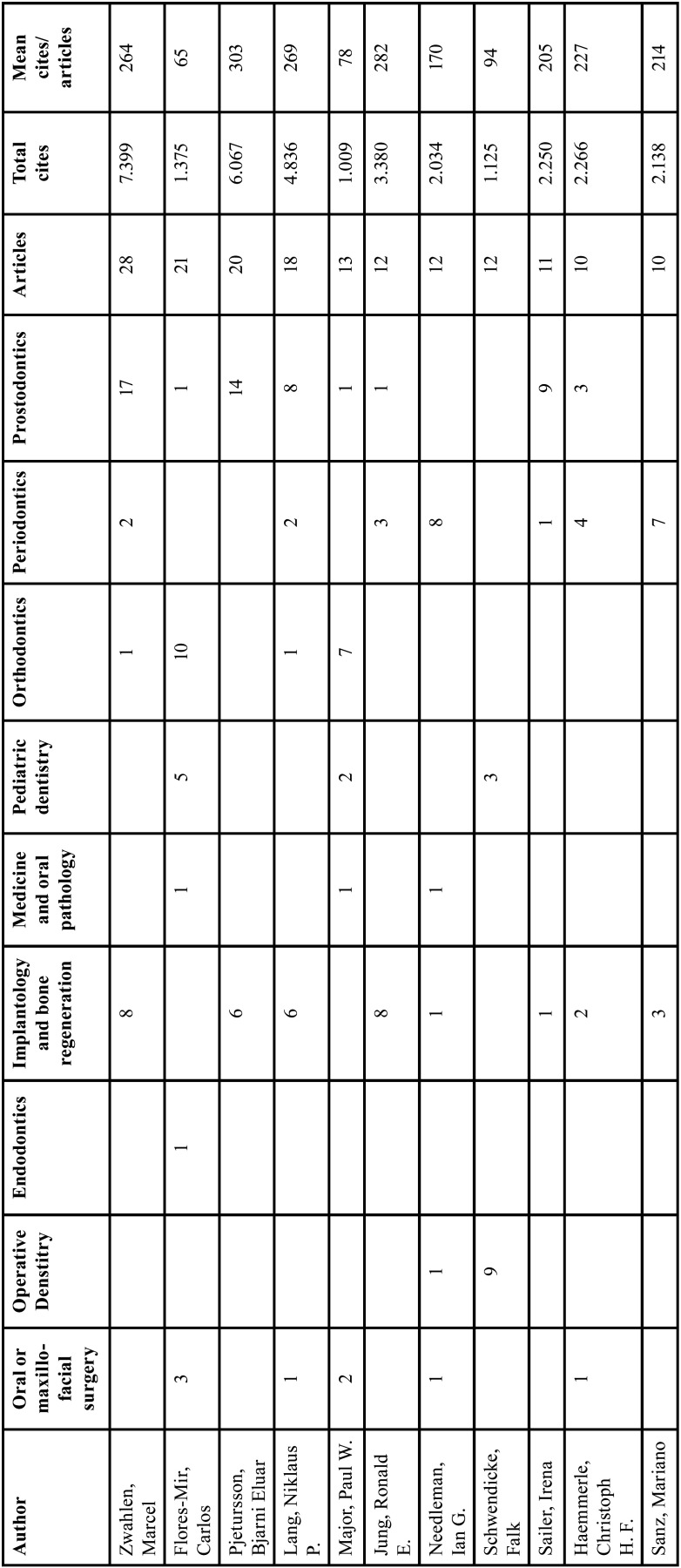
Most productive authors with more than 10 published works.

If we sort the authors by the total number of citations of their most-cited articles, Marcel Zwahlen continues to be the most cited (7,399 citations), followed by Bjarni Eluar Pjetursson (6,067 citations). However, if we sort them by the average number of citations of the articles, then JM Hirsch and U Lekholm, authors of two articles in the field of Periodontics, have the highest average of 839 citations.

Table 4 shows the most productive institutions, all of which belong to developed European countries, the USA, and China. This classification hardly changes the ranking when sorted by the number of citations. When sorted by the number of citations per article, the first place is occupied by the Seattle Children Hospital (USA), with an average of 475 citations per article, followed by Uppsala University (Sweden) with 450 citations. The university with the most publications is the University of Bern (Switzerland); this is an institution, which together with the University of Gothenburg (Sweden), has the first position with respect to the average citations per article.

**Table 4:**
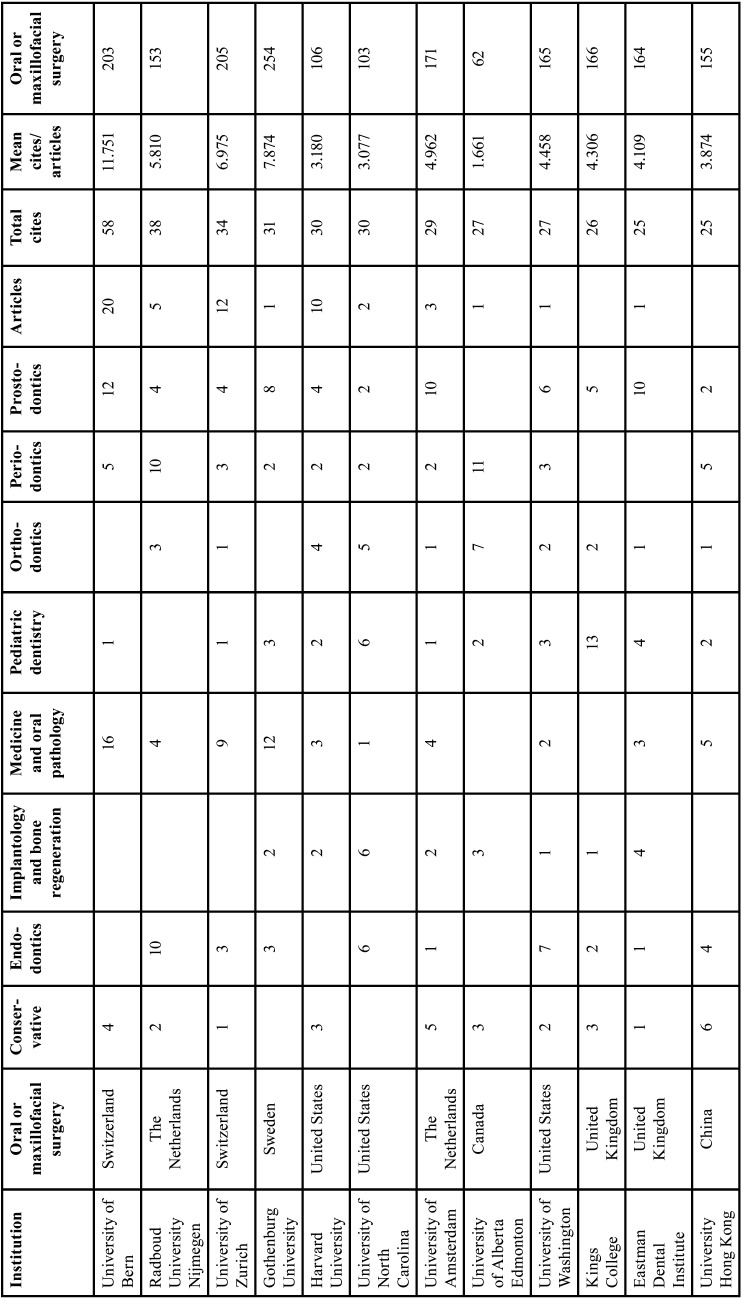
Most productive institutions with more than 25 articles published.

Finally, when analysing the most productive countries, economically powerful countries, such as the USA, the UK, and Switzerland head the list. However, countries such as Iceland and Singapore lead the ranking of citations per work, with more than 200 citations of their studies (Table 5).

**Table 5:**
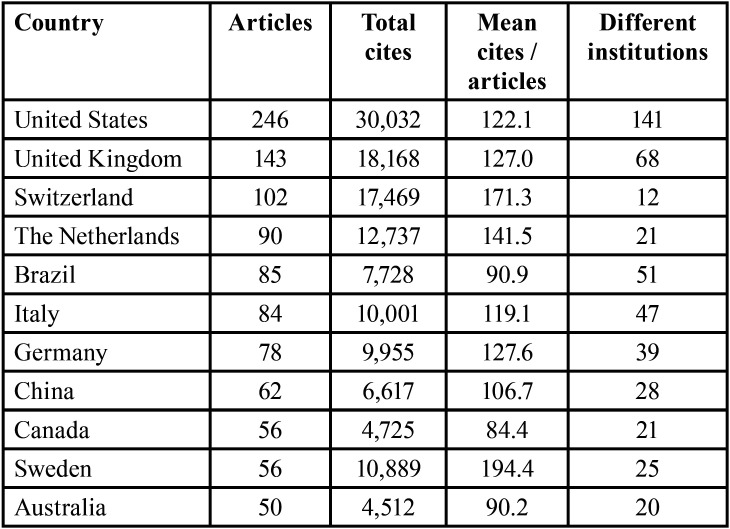
Most productive countries with more than 50 published works.

Regarding the type of review performed, it is quite common in all specialties to conduct reviews that do not include statistical analysis, i.e. reviews of the literature or systematic reviews. Specifically, the most common type of review in all specialties is the systematic review, followed by systematic reviews with meta-analysis. The only exception is in Periodontics, where literature reviews take second place.

Another noteworthy fact is that Prosthodontics is the specialty with the most systematic reviews, accounting for 80.48% of the scientific production. The specialty with the most published systematic reviews with meta-analysis is Paediatric dentistry (42.68%) and that with the most published reviews is Periodontics (27.35%) (Table 6).

**Table 6:**
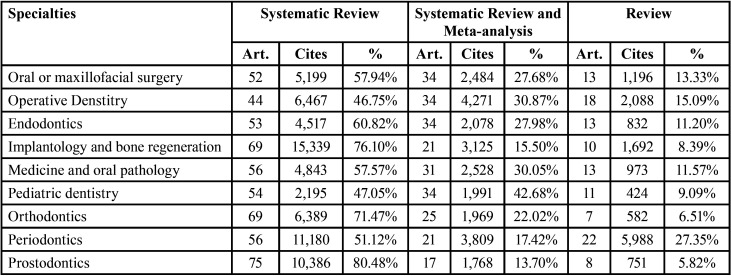
Distribution of the review typology by specialties.

Regarding reviews, regardless of the specialty to which they belong, the most frequent subject was found to be cancer, accounting for 50 of the 900 existing papers. The other major subject areas were those related to dental pathology and therapeutics, prosthetic issues, implantology issues, and periodontal issues (Table 7).

**Table 7:**
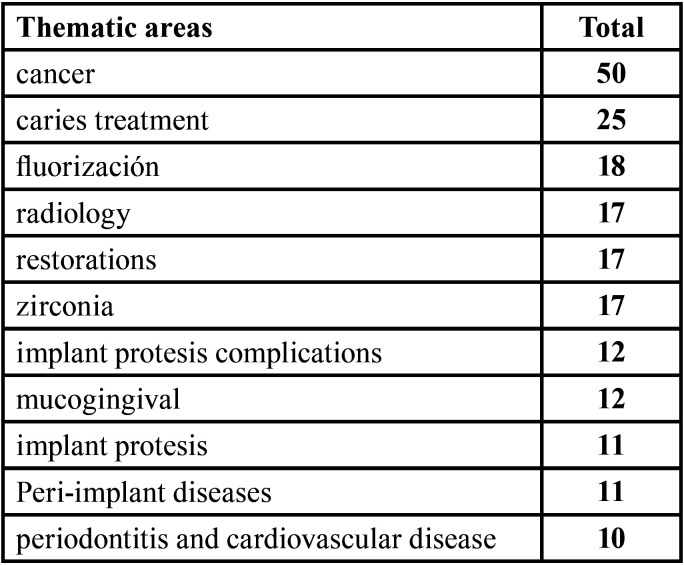
Thematic areas with more than 10 articles.

Regarding the funding of the studies, 198 of the 903 works received financing from 213 institutions in 27 different countries.

It can be observed that funding is most commonly acquired from private companies, followed by the national government and public universities (Table 8).

**Table 8:**
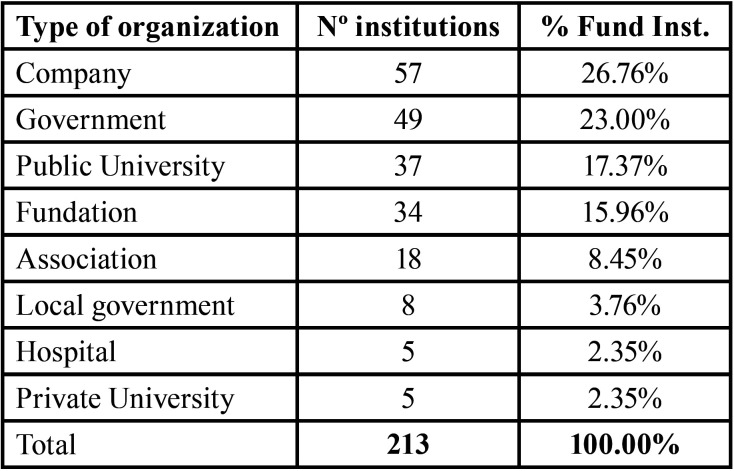
Funding institutions.

Furthermore, Periodontics received the most funding, followed by the specialties of Dental Pathology and Therapeutics and Medicine and Oral Pathology (Table 9). The country that finances the most by far is the USA.

**Table 9:**
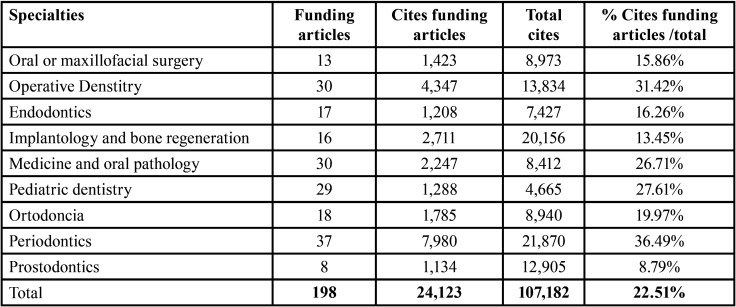
Distribution of funding by specialties and number of citations of the funded articles.

In total, 900 articles have been published in 69 journals, considering only the area of dentistry. It was observed that those receiving more citations tended to be better ranked according to the Journal Citation Reports (JCR) index. The journal receiving the most citations is the Clinical Journal of Periodontology, which is also the one that has published the most articles. However, the journal with the highest number of citations per article is the International Journal of Periodontics and Restorative Dentistry, with only two published articles ( Table 10).

**Table 10:**
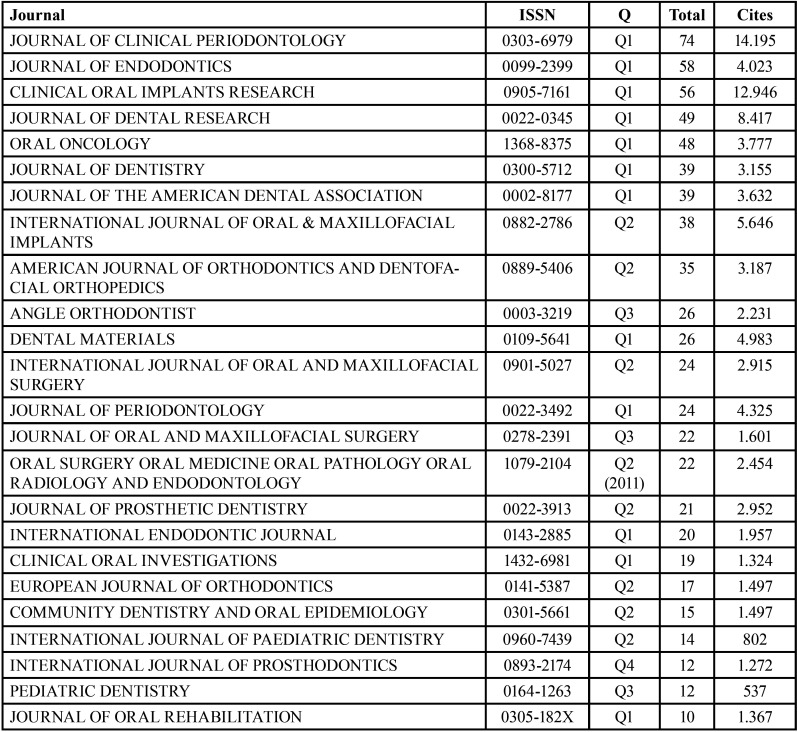
List of journals with 10 or more articles, last year quartile (Q), number of articles and citations.

## Discussion

When studying temporal evolution, there is an increasing trend in scientific production as the years go by. This tendency toward exponential growth of publications can be observed in bibliometric studies on scientific production in areas such as Implantology (5), Periodontics (8), Dental Pathology and Therapeutics (15), and Oral Medicine (16) . This trend has also been observed in reviews (2).

The obvious explanation for this upward trend in the number of publications is the increase in scientific production, especially in the case of systematic reviews, which is one of the most cited article types. This is possibly due to their important place in providing scientific evidence as well as their ability to synthesise the covered topics.

It has been shown that the majority of publications do not include statistical studies and that systematic reviews are the most frequent article type, followed by systematic reviews with meta-analysis and literature reviews. Although there are certain areas (Periodontics) that do not follow this trend, it has also been observed in other studies (2,17). This could be because in this specialty, we find a large number of consensus documents that are published in high-impact journals, thus receiving a large number of citations.

Regarding the analysis according to specialties, the two specialties that stand out by far in the number of total citations and number of citations per article are Periodontics and Implantology & Bone Regeneration. This is related to the position that journals of these specialties hold in the JCR ranking; this trend has also been observed in similar other publications(2). Furthermore, these specialties have been publishing reviews for many years, as mentioned previously; therefore, they have had more time to receive citations, unlike Paediatric dentistry, which has had substantial number of reviews published only in the last decade and that could be the reason for fewer citations.

However, despite this trend in specialties, the specific topic with the highest number of publications is oncology. This is because it is the recurring topic in specialties such as Oral Medicine and Oral and Maxillofacial Surgery.

The most productive institutions were from developed European countries, the USA, and China. The University of Bern (Switzerland) and University of Gothenburg (Sweden) had the highest average citations per article, both of which participated in the inception of Implantology. Hence, they are two great centres of scientific production and are highly regarded for their level of citations.

As seen in other bibliometric studies(8,18), the country that stands out the most in terms of scientific production is the USA. This is a fairly frequent finding in other bibliometric studies(5,8). Undoubtedly, an influencing factor is the large size of this country, and consequently, the number of centres and researchers. For the same reasons, it leads the ranking of countries with respect to the highest funding support, as shown in previous bibliometric studies on implants(18).

## Conclusions

Together with an exponential increase in the number of publications in dentistry, there has been an increase in the number of publications in systematic reviews. The areas publishing the most articles and having the most citations are Periodontics and Implantology, despite the fact that the most studied topic is cancer.
